# Supplementary material for: Clinical Validation of a Size-Based Microfluidic Device for Circulating Tumor Cell Isolation and Analysis in Renal Cell Carcinoma
Source: Int J Mol Sci. 2023 May 7;24(9):8404. doi: 10.3390/ijms24098404 (PMC10178884; doi:10.3390/ijms24098404)
Supplement: Supplementary file 1 [file ijms-24-08404-s001.zip › Document S1.pdf]

SUPLEMENT S1. CORRELATION ANALYSIS CHARTS

Correlation analysis for group M0

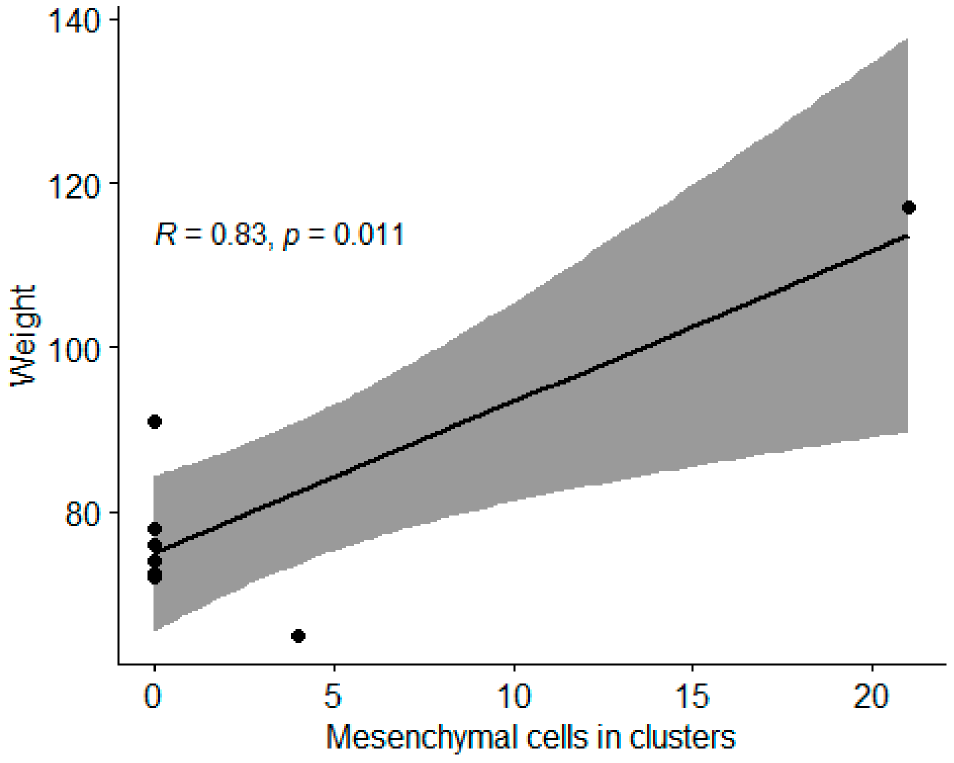

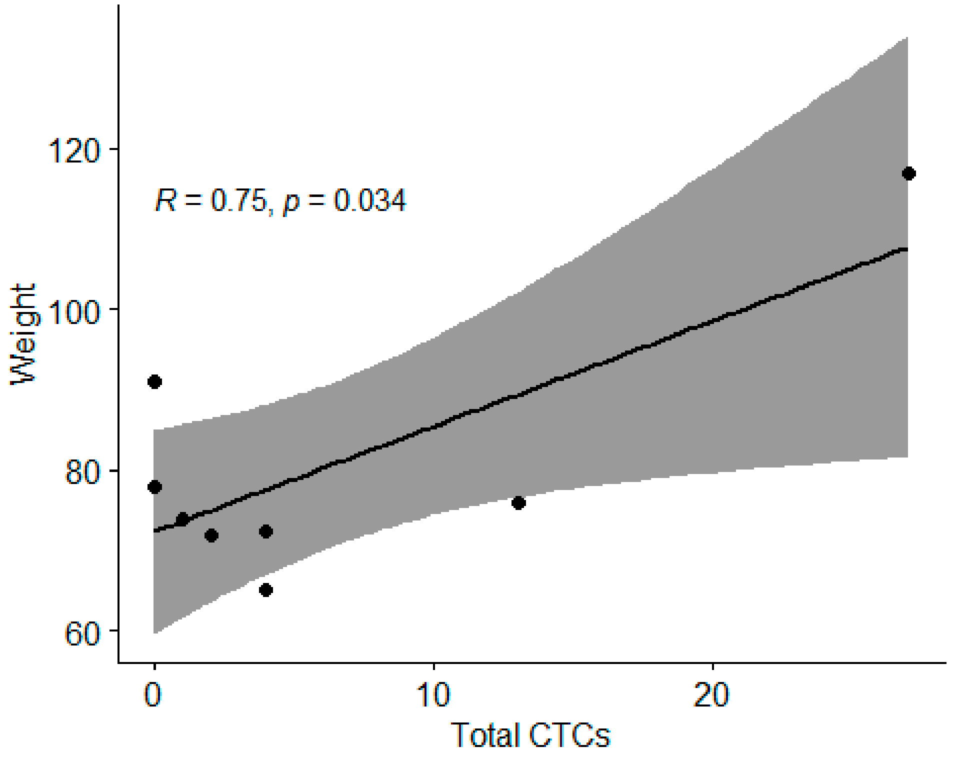

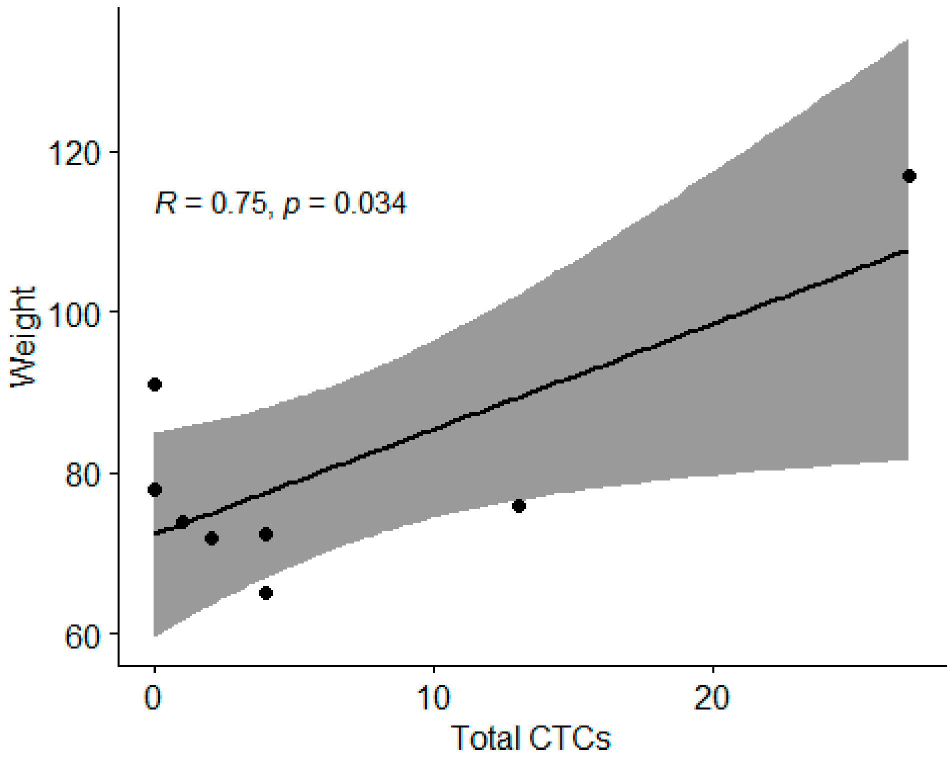

R = 0.75, p = 0.034
Weight
120
100
80
60
0
10
20
Total CTCs

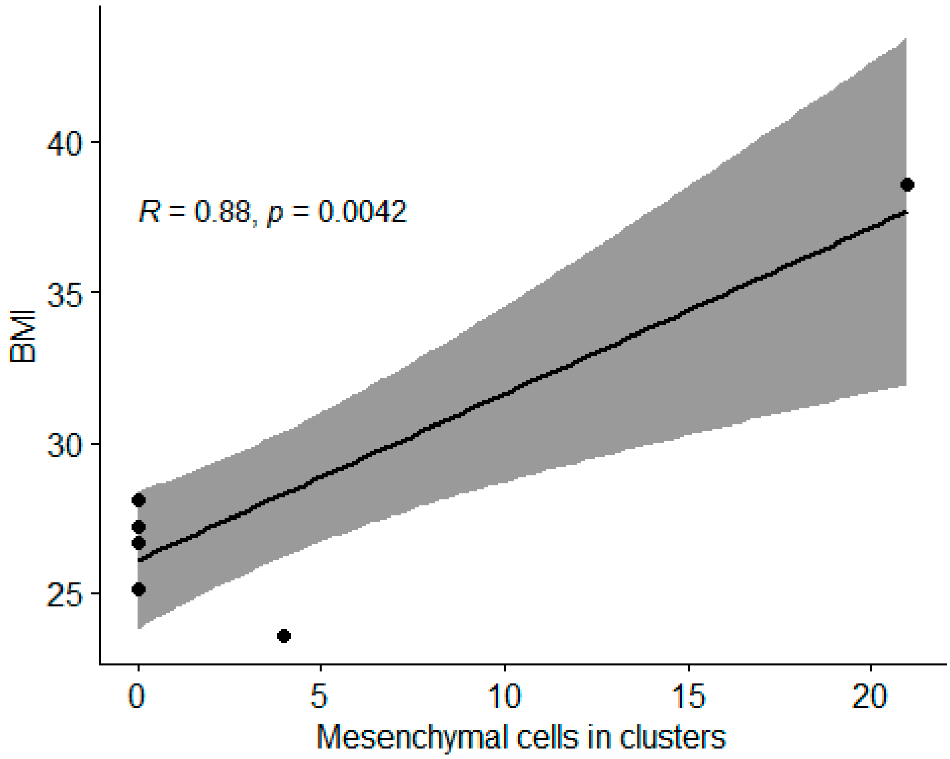

R = 0.88, p = 0.0042
BMI
40
35
30
25
0
5
10
15
20
Mesenchymal cells in clusters

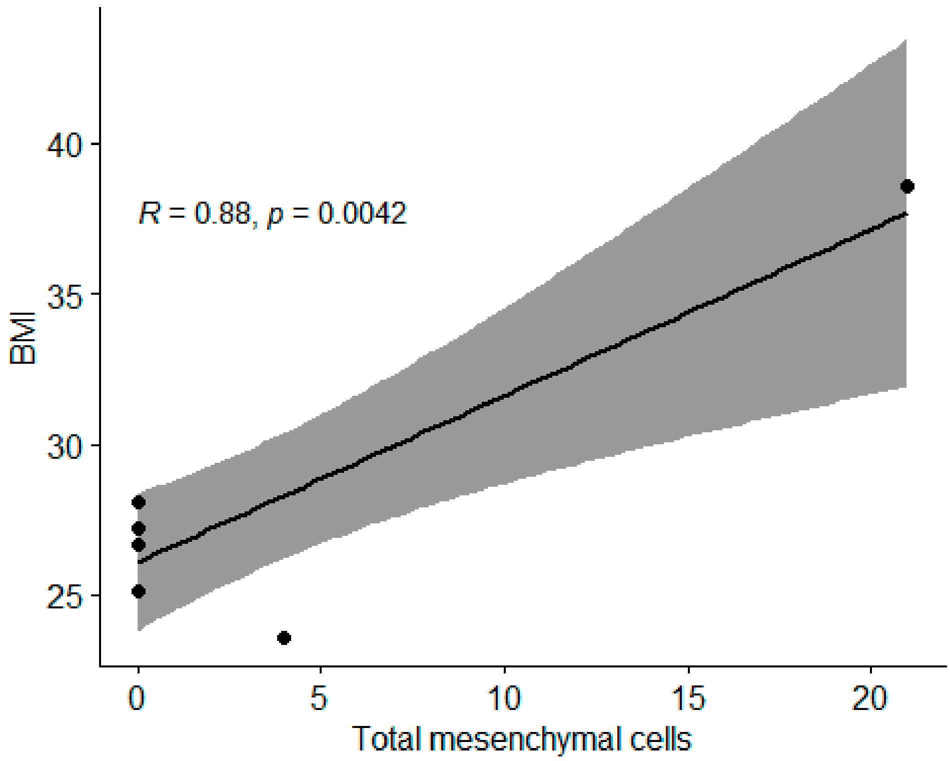

R = 0.88, p = 0.0042
BMI
40
35
30
25
0
5
10
15
20
Total mesenchymal cells

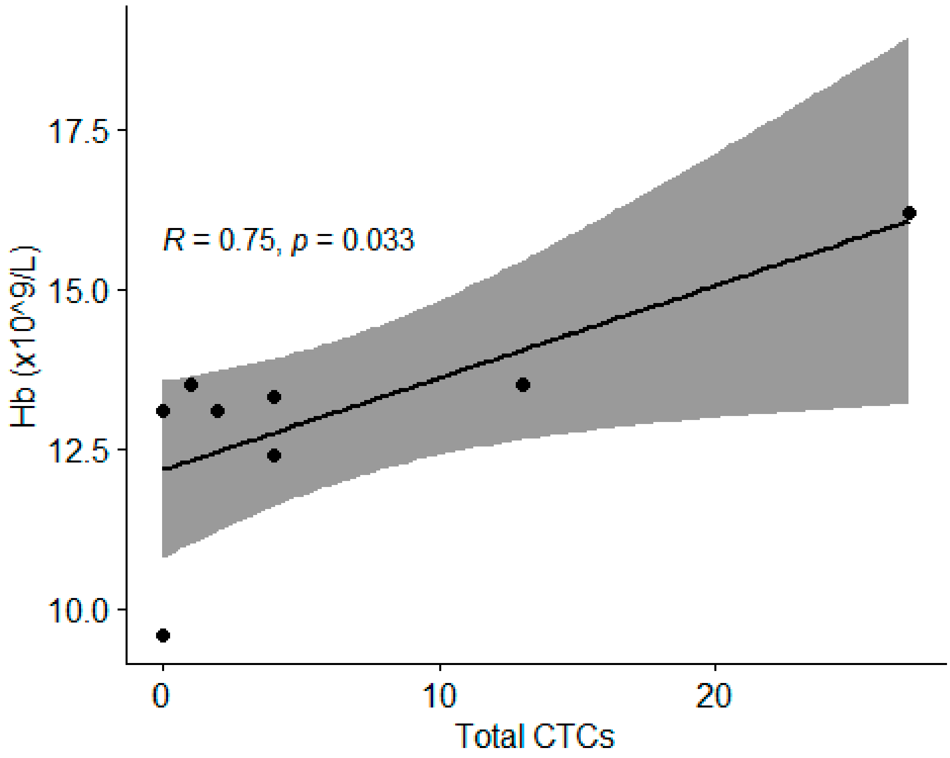

R = 0.75, p = 0.033
Hb (x10^9/L)
17.5
15.0
12.5
10.0
0
10
20
Total CTCs

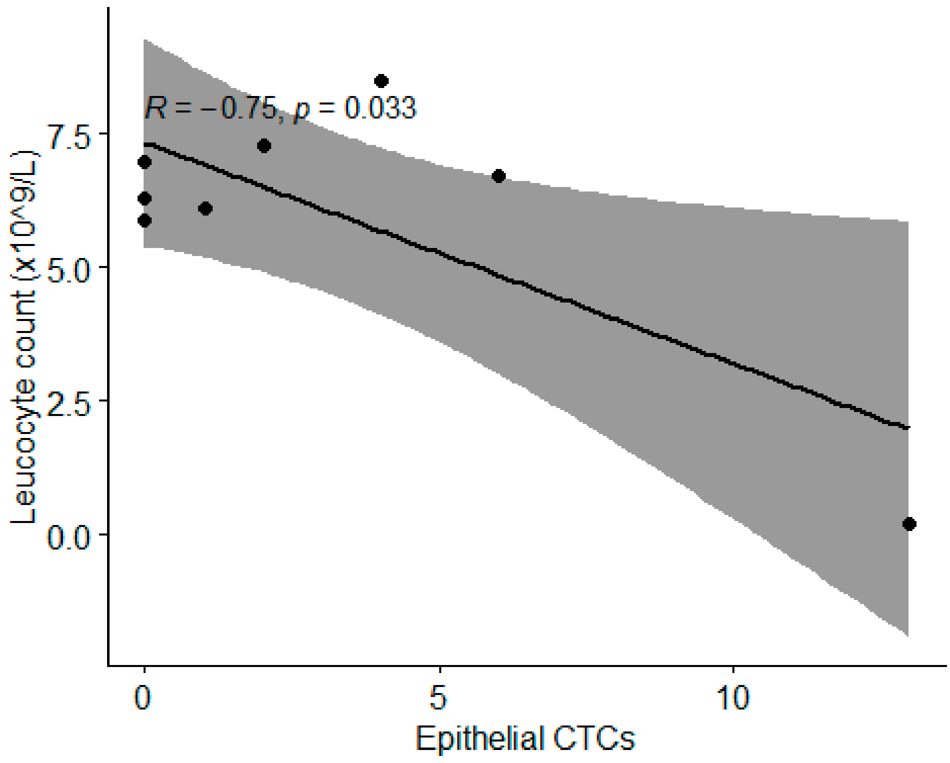

R = −0.75, p = 0.033
Leucocyte count (x10^9/L)
7.5
5.0
2.5
0.0
0
5
10
Epithelial CTCs

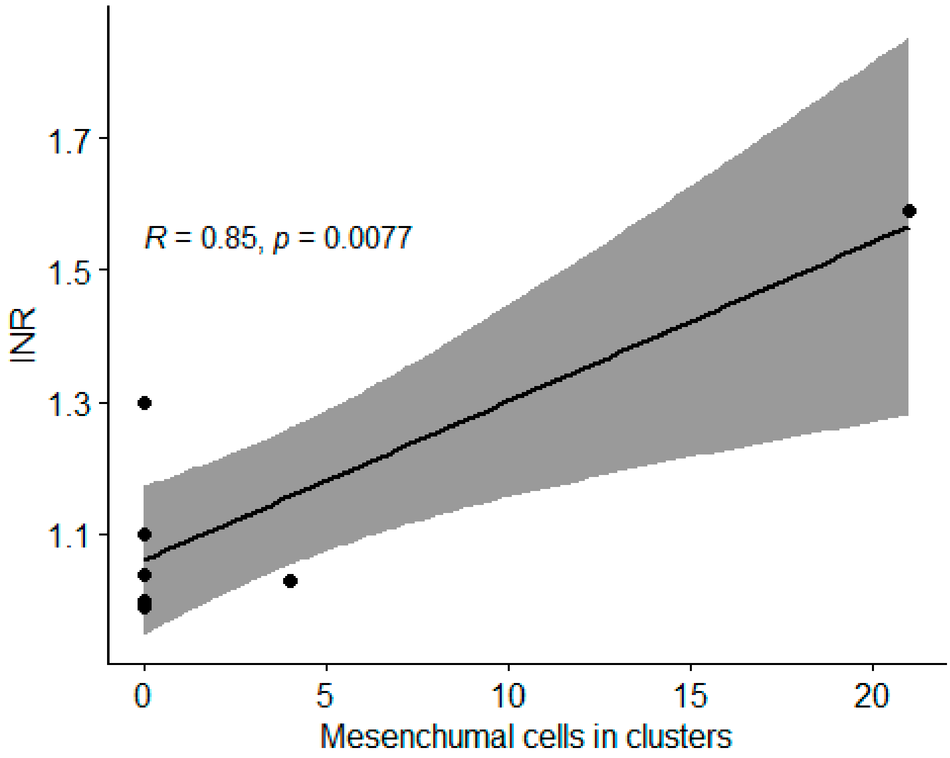

R = 0.85, p = 0.0077
INR
1.7
1.5
1.3
1.1
0
5
10
15
20
Mesenchumal cells in clusters

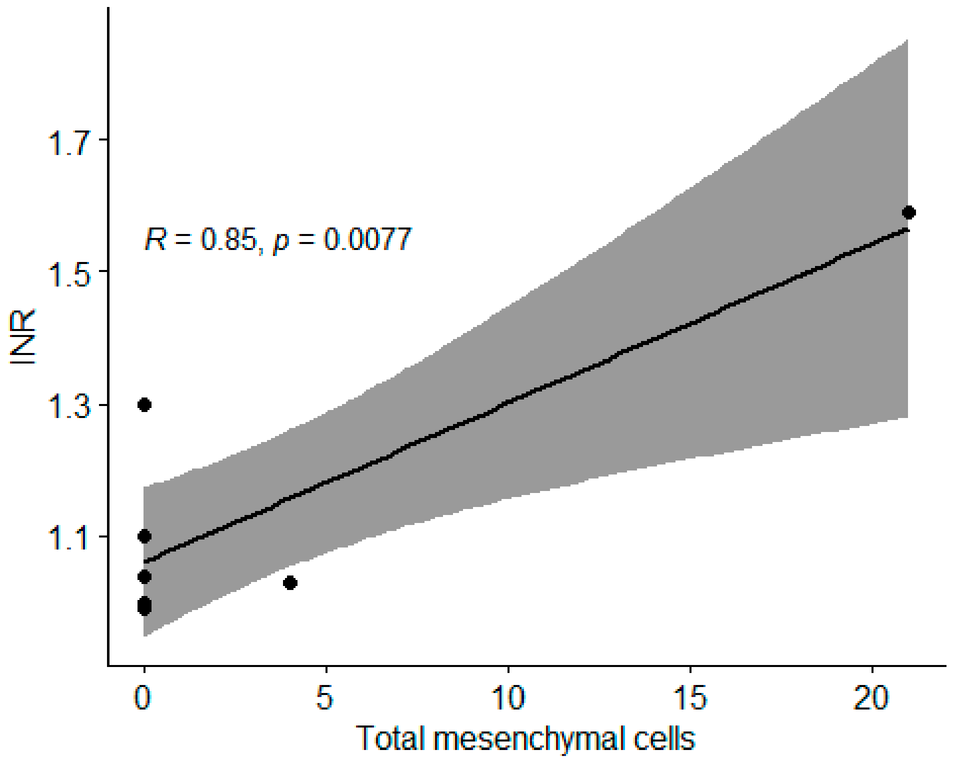

R = 0.85, p = 0.0077
1.7
1.5
1.3
1.1
INR
0
5
10
15
20
Total mesenchymal cells

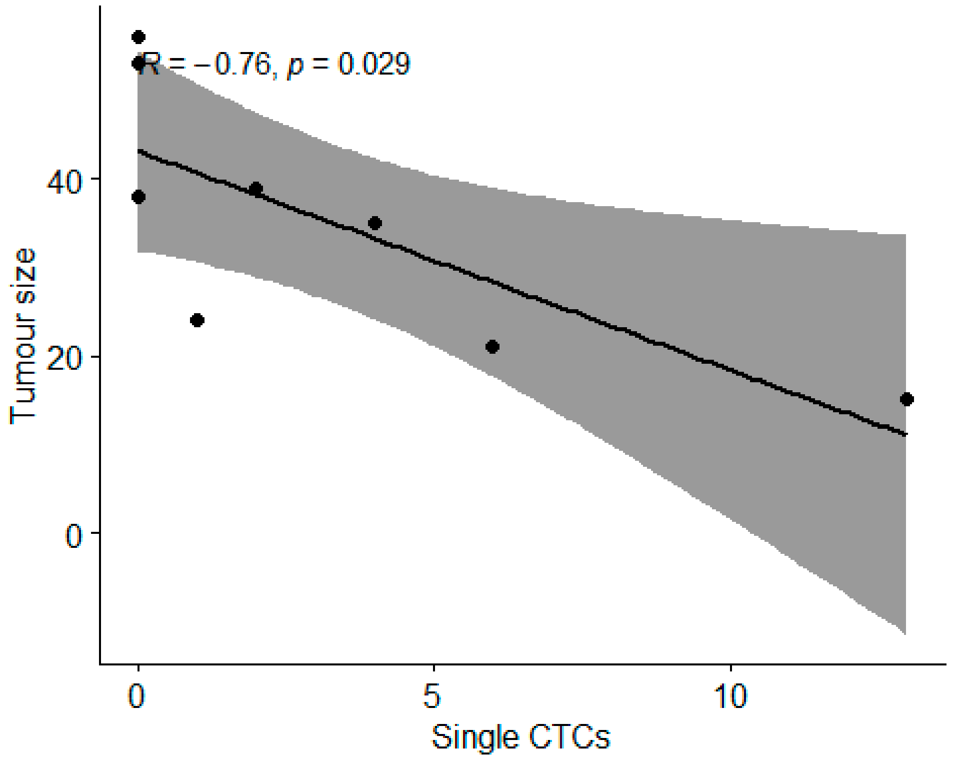

R = −0.76, p = 0.029
40
20
0
Tumour size
0
5
10
Single CTCs

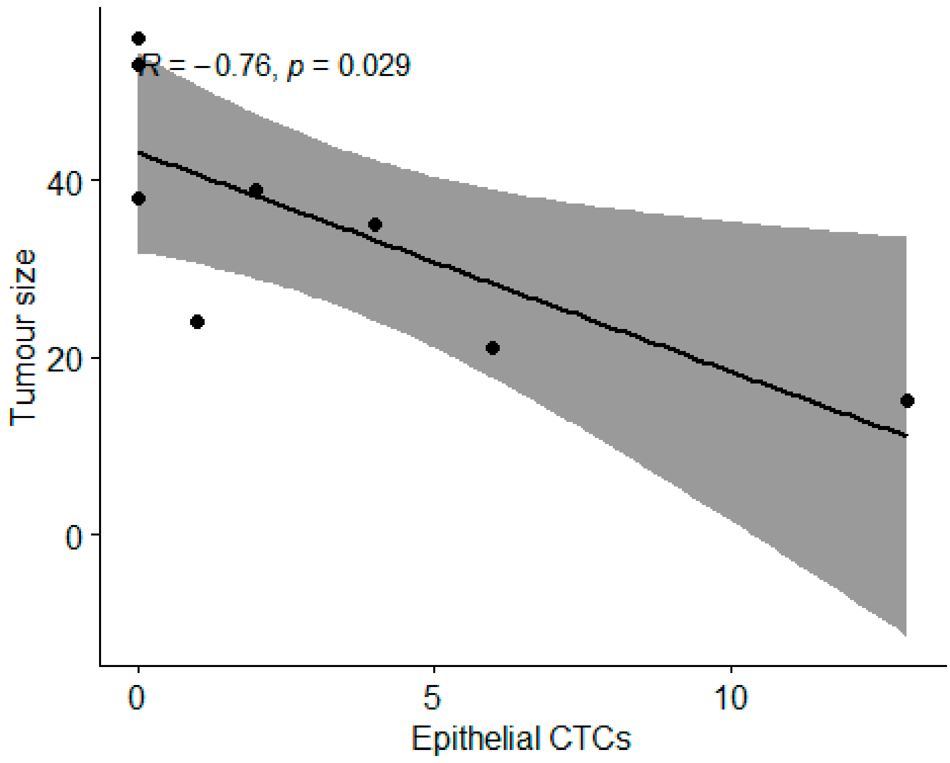

Correlation analysis for group M1

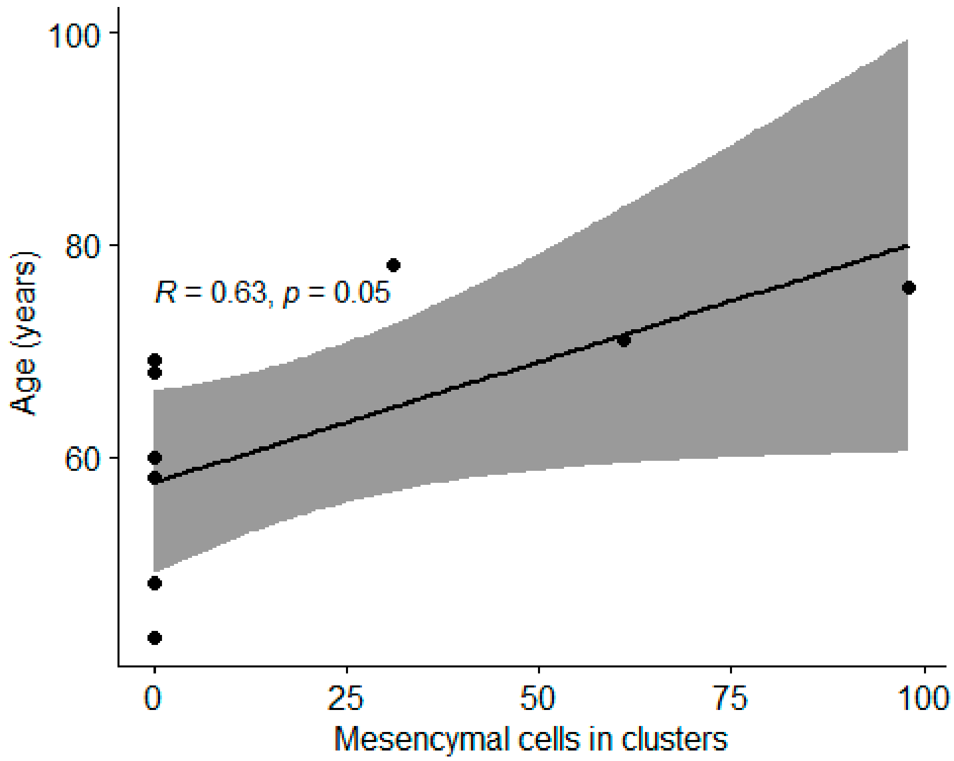

Age (years)
100
80
60
R = 0.63, p = 0.05
0
25
50
75
100
Mesencymal cells in clusters

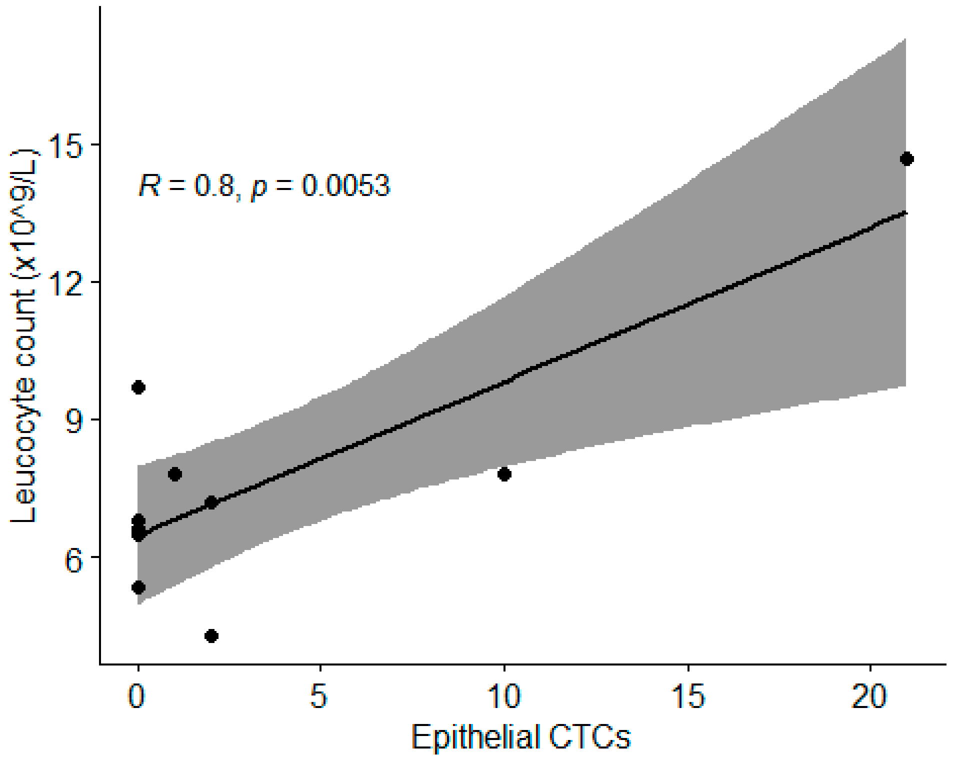

Leucocyte count (x10^9/L)
15
12
9
6
R = 0.8, p = 0.0053
0
5
10
15
20
Epithelial CTCs

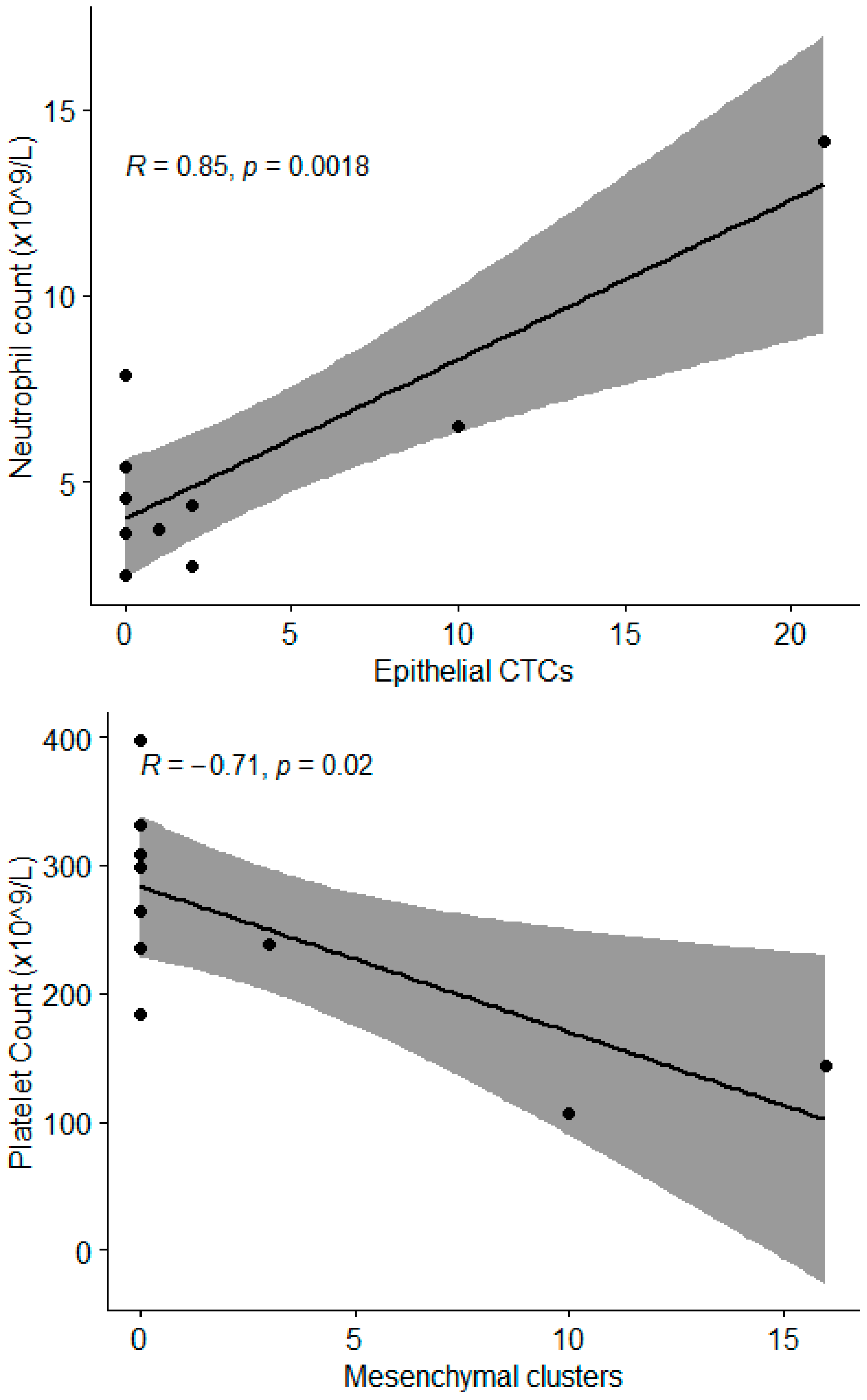

R = 0.85, p = 0.0018
Neutrophil count (x10^9/L)
15
10
5
0
5
10
15
20
Epithelial CTCs
R = −0.71, p = 0.02
Platelet Count (x10^9/L)
400
300
200
100
0
0
5
10
15
Mesenchymal clusters

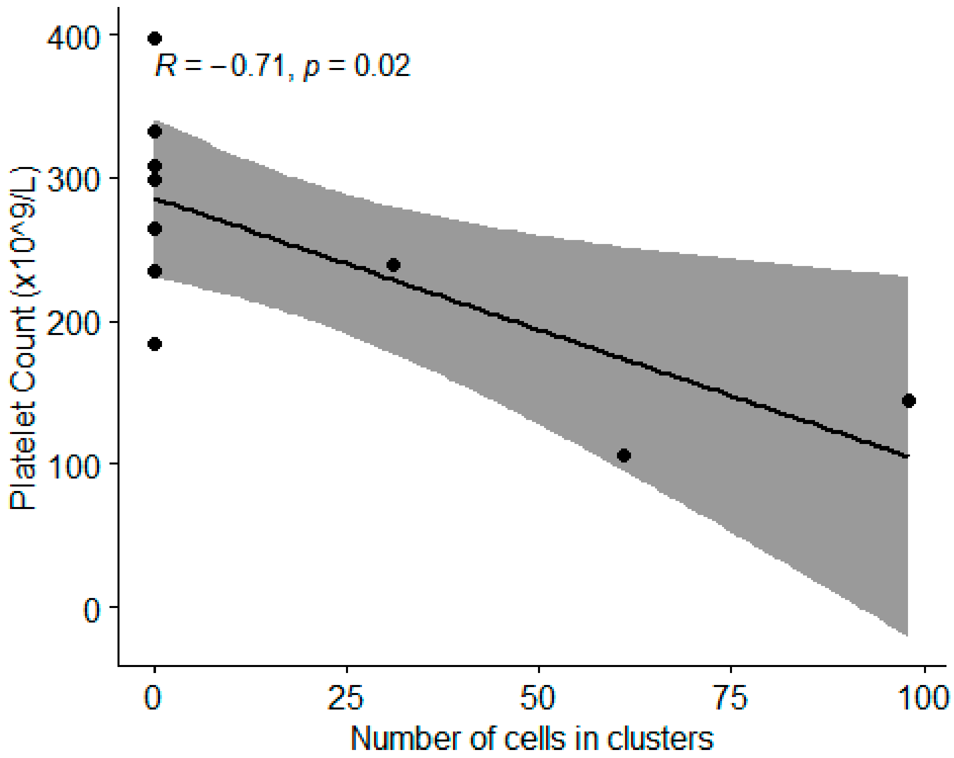

400
300
200
100
0
Platelet Count (x10^9/L)
R = −0.71, p = 0.02
0
25
50
75
100
Number of cells in clusters

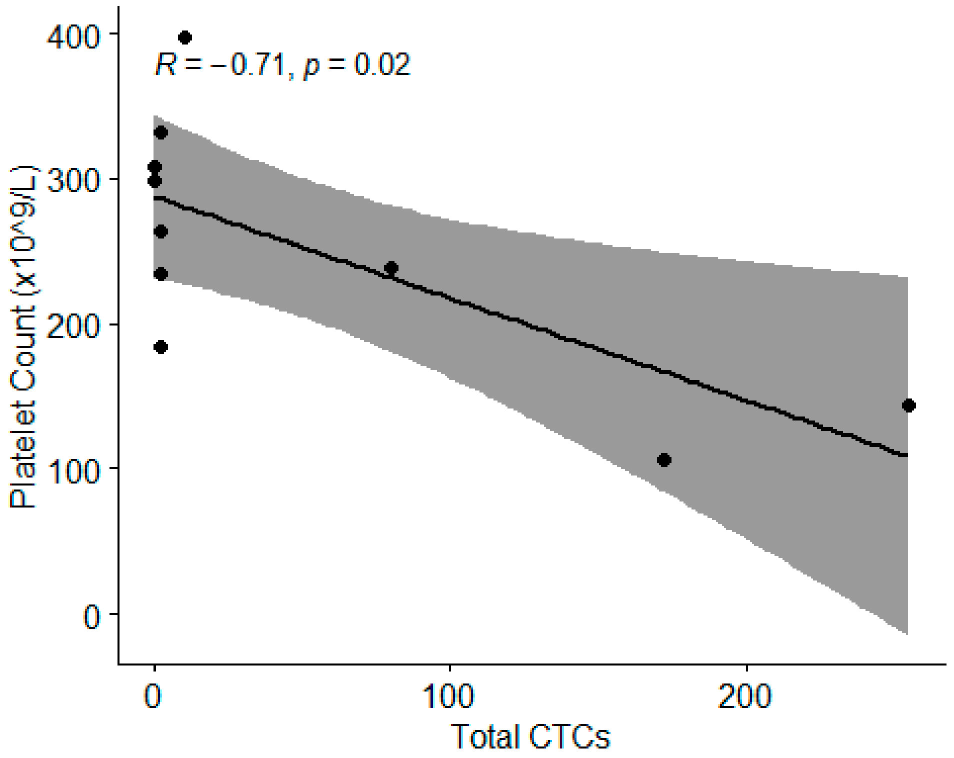

400
300
200
100
0
Platelet Count (x10^9/L)
R = −0.71, p = 0.02
0
100
200
Total CTCs

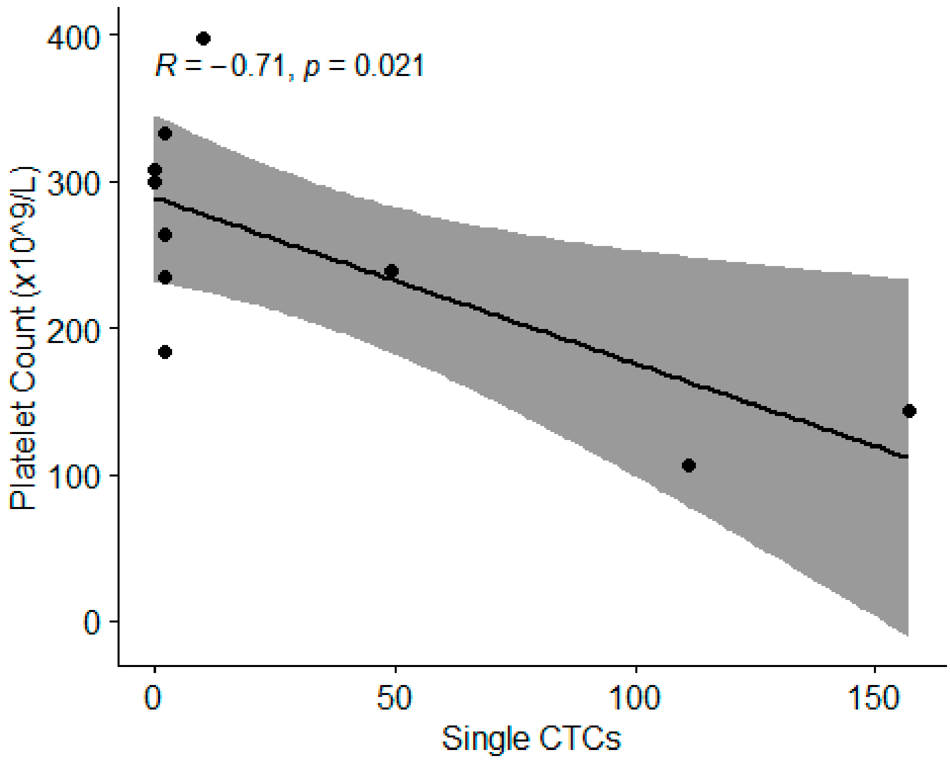

R = −0.71, p = 0.021
Platelet Count (x10^9/L)
Single CTCs

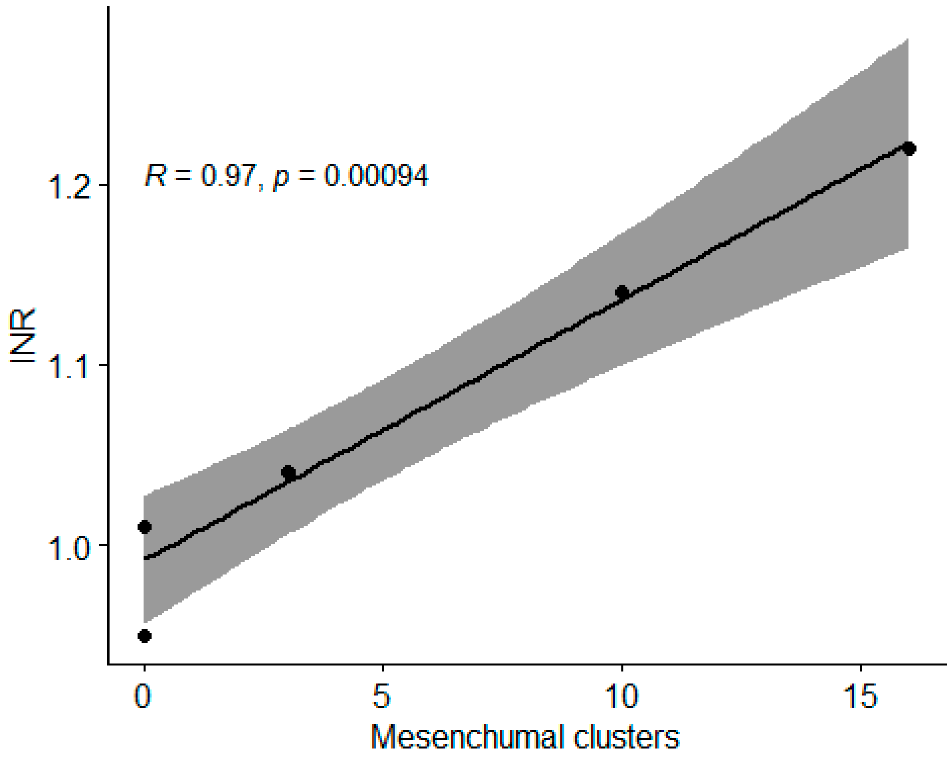

R = 0.97, p = 0.00094
INR
Mesenchumal clusters

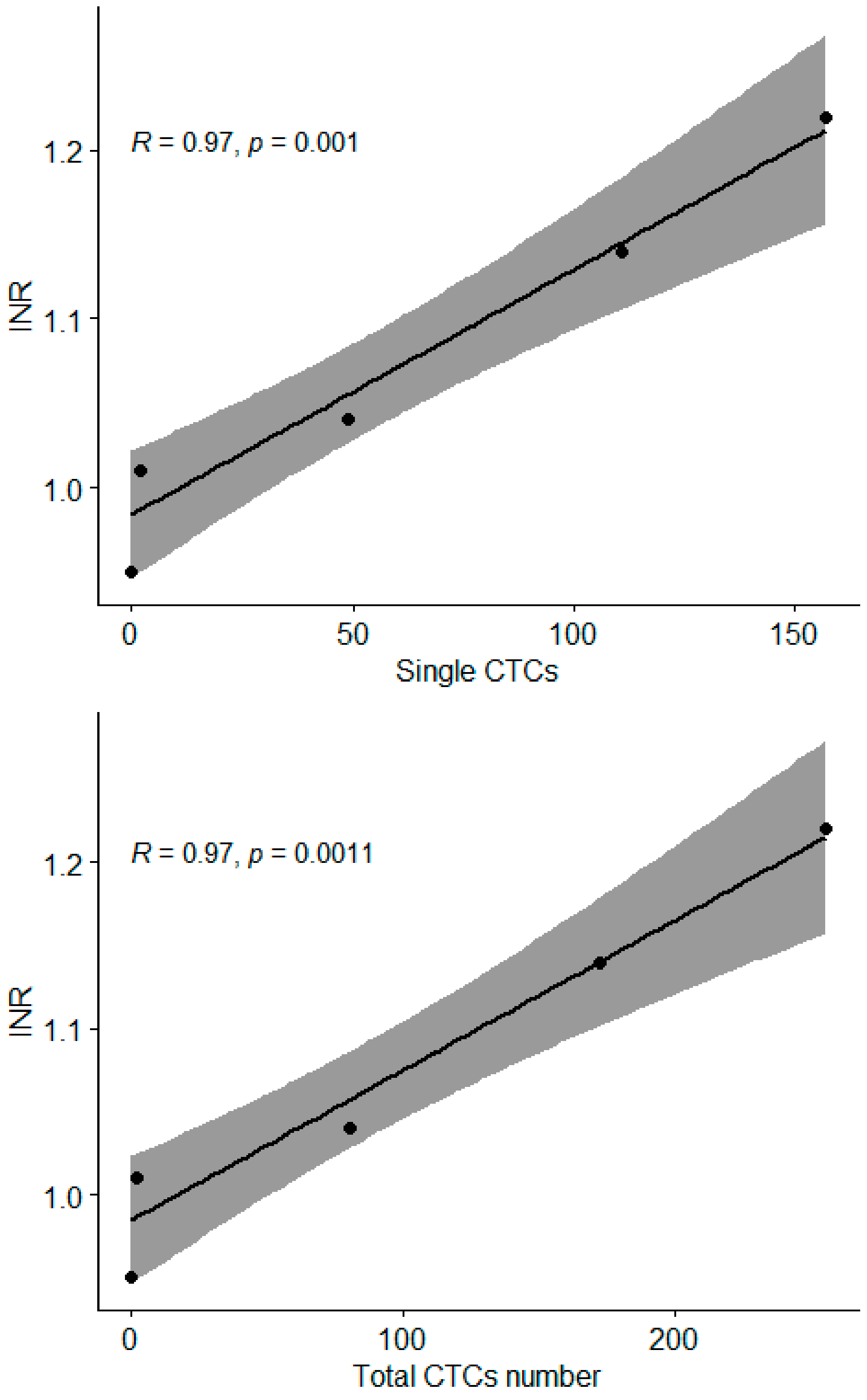

R = 0.97, p = 0.001
1.2
1.1
1.0
INR
0
50
100
150
Single CTCs
R = 0.97, p = 0.0011
1.2
1.1
1.0
INR
0
100
200
Total CTCs number

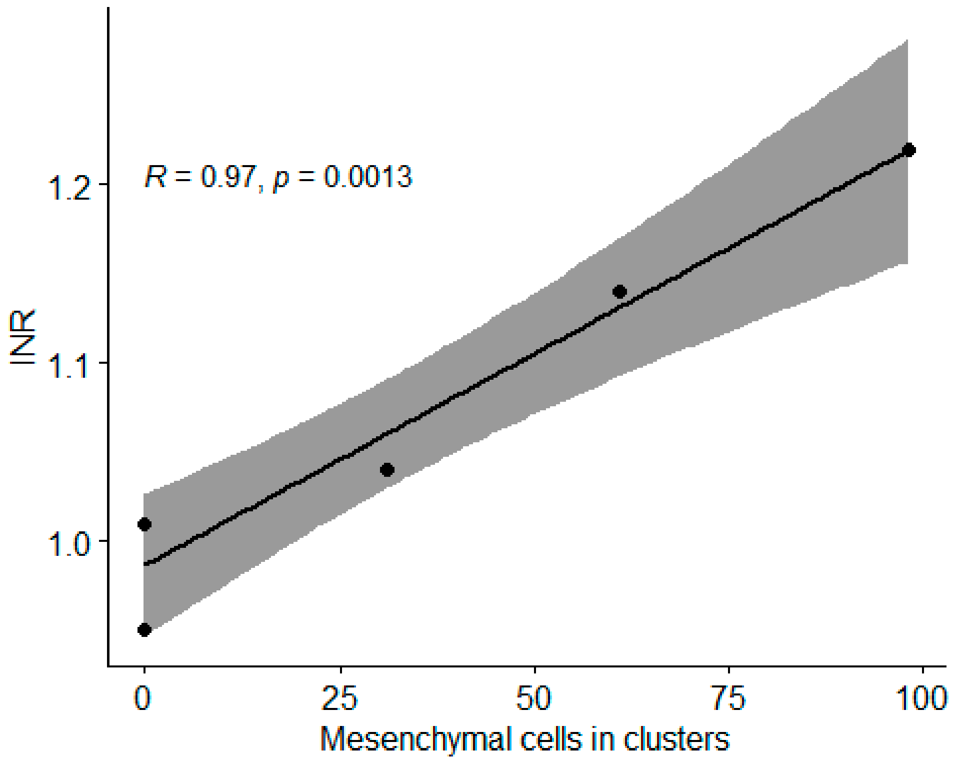

R = 0.97, p = 0.0013
INR
1.2
1.1
1.0
0
25
50
75
100
Mesenchymal cells in clusters

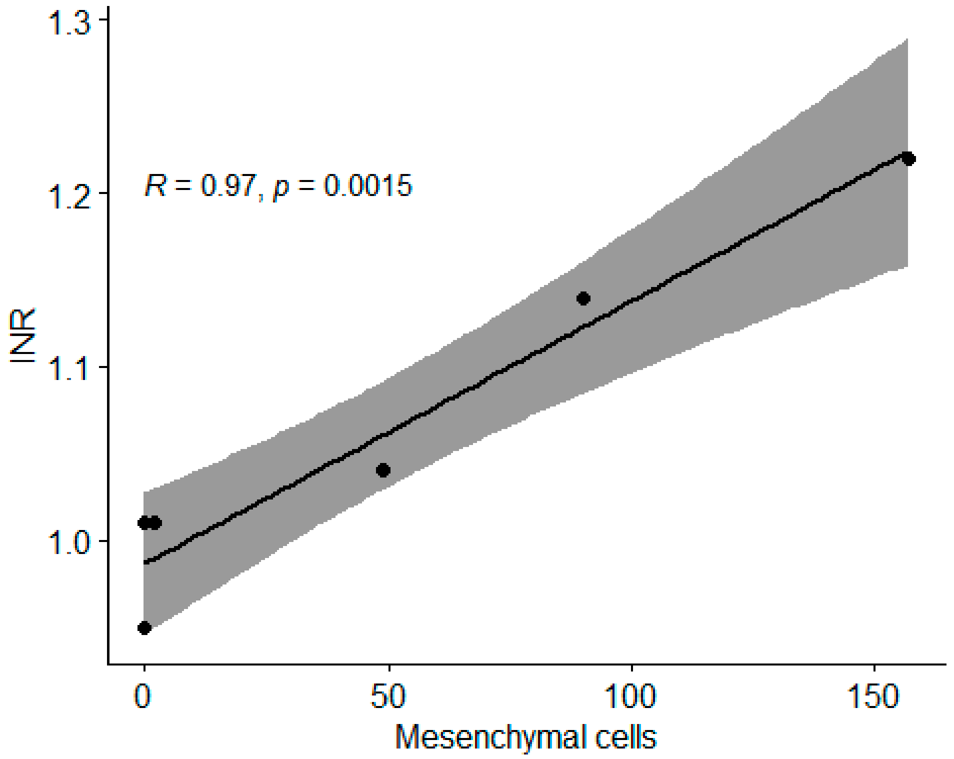

R = 0.97, p = 0.0015
INR
1.3
1.2
1.1
1.0
0
50
100
150
Mesenchymal cells

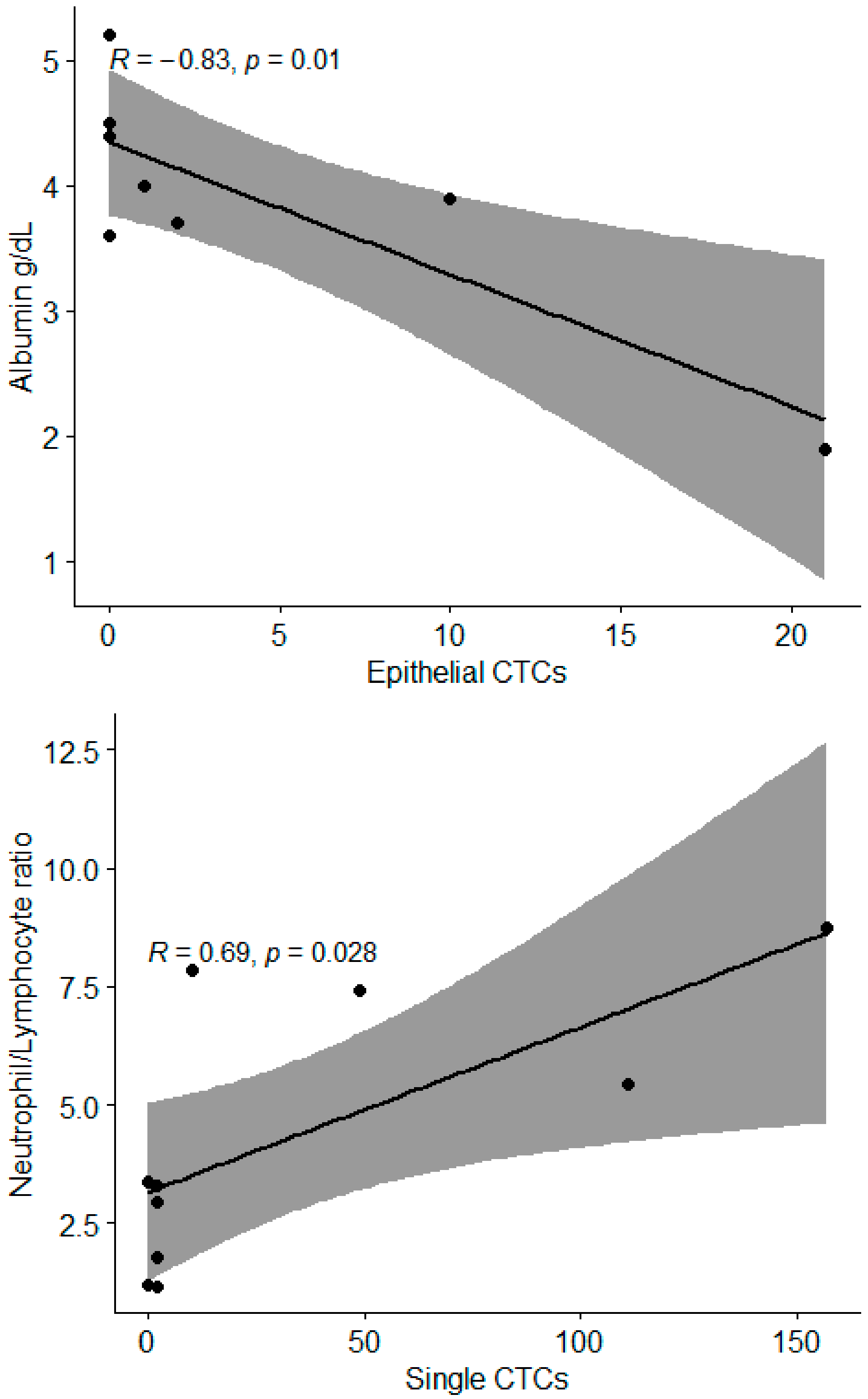
R = −0.83, p = 0.01
Albumin g/dL
5
4
3
2
1
0
5
10
15
20
Epithelial CTCs
R = 0.69, p = 0.028
Neutrophil/Lymphocyte ratio
12.5
10.0
7.5
5.0
2.5
0
50
100
150
Single CTCs

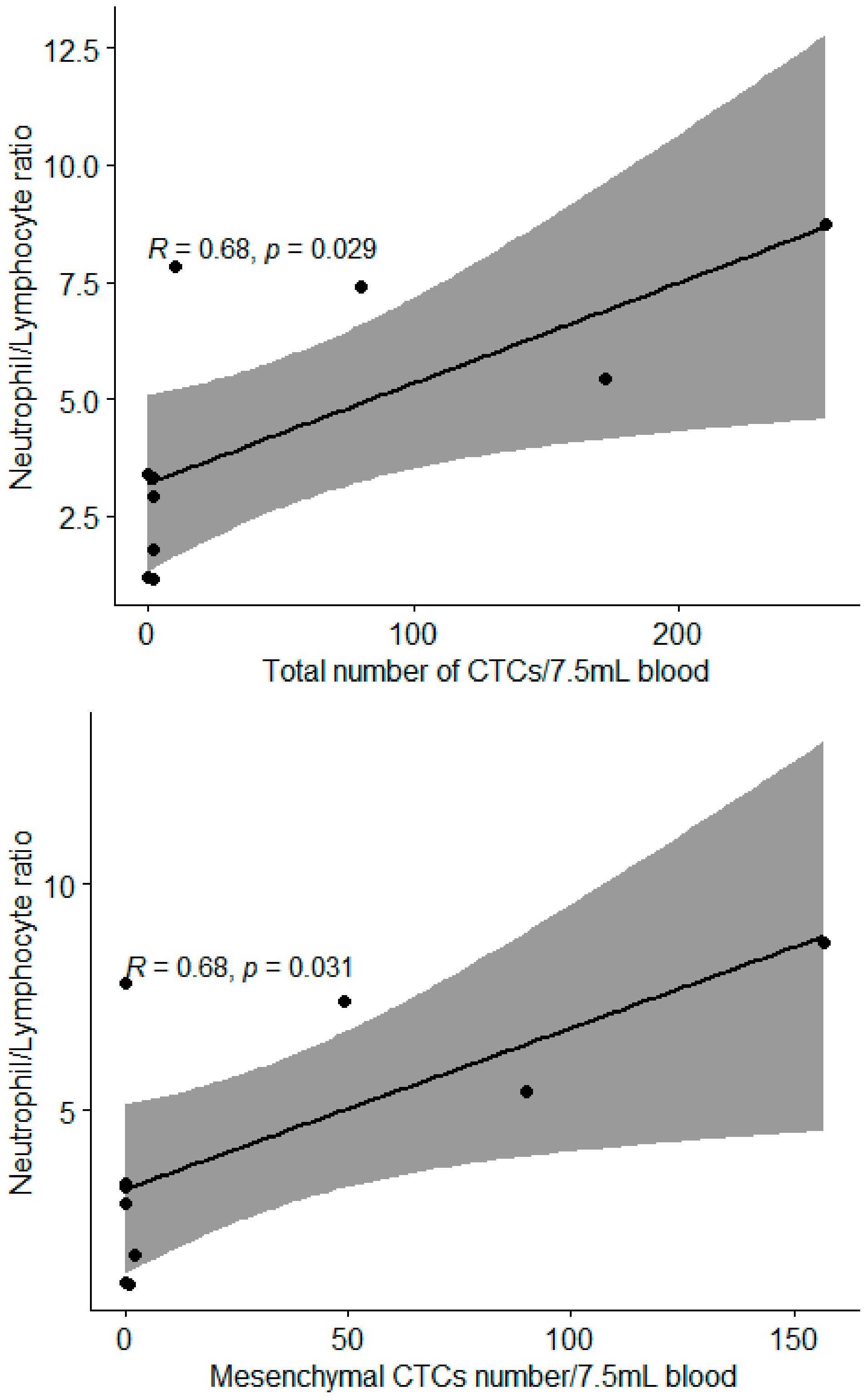

Neutrophil/Lymphocyte ratio
12.5
10.0
7.5
5.0
2.5
R = 0.68, p = 0.029
0
100
200
Total number of CTCs/7.5mL blood
Neutrophil/Lymphocyte ratio
10
5
R = 0.68, p = 0.031
0
50
100
150
Mesenchymal CTCs number/7.5mL blood

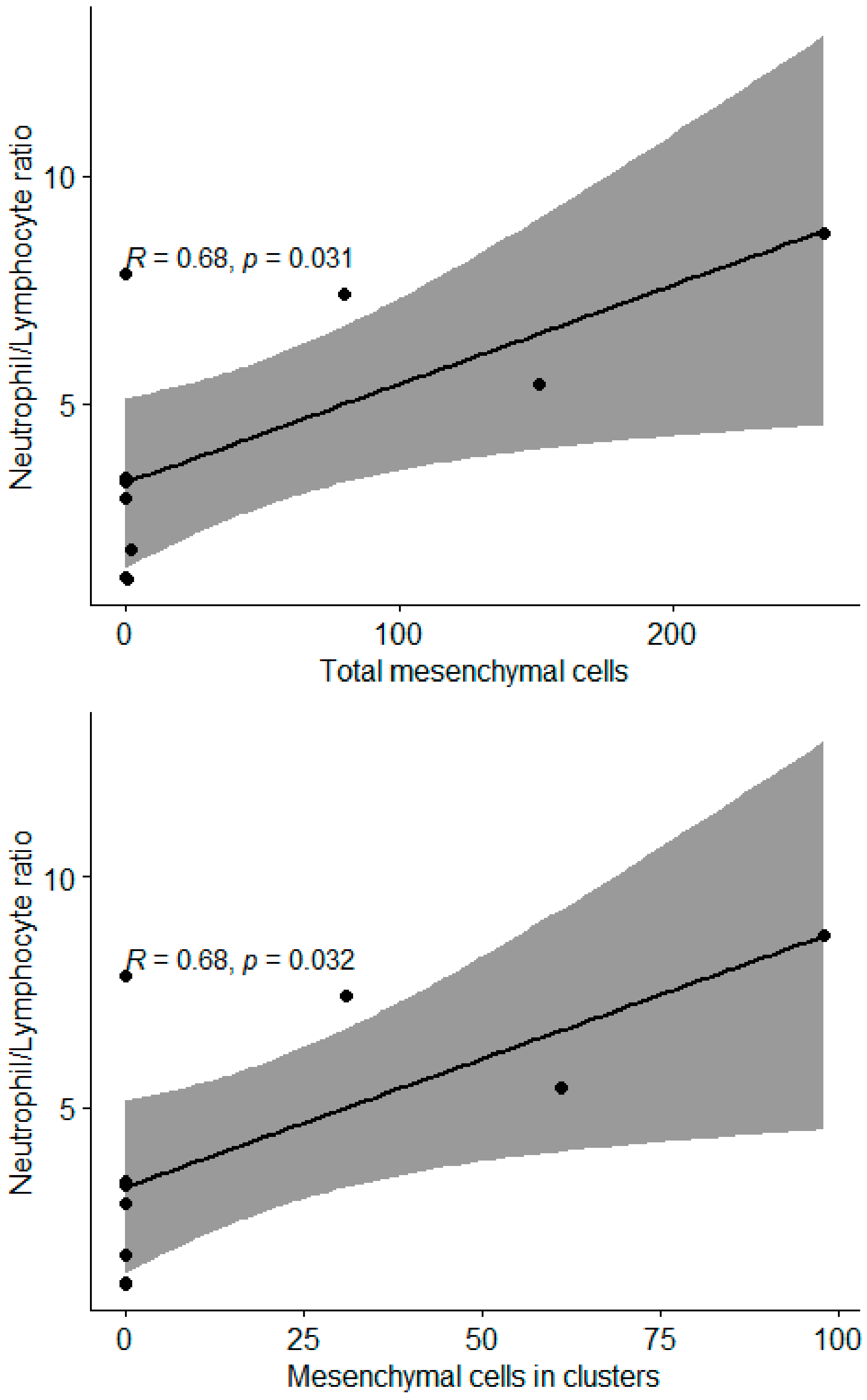

Neutrophil/Lymphocyte ratio
10
5
R = 0.68, p = 0.031
0
100
200
Total mesenchymal cells
Neutrophil/Lymphocyte ratio
10
5
R = 0.68, p = 0.032
0
25
50
75
100
Mesenchymal cells in clusters

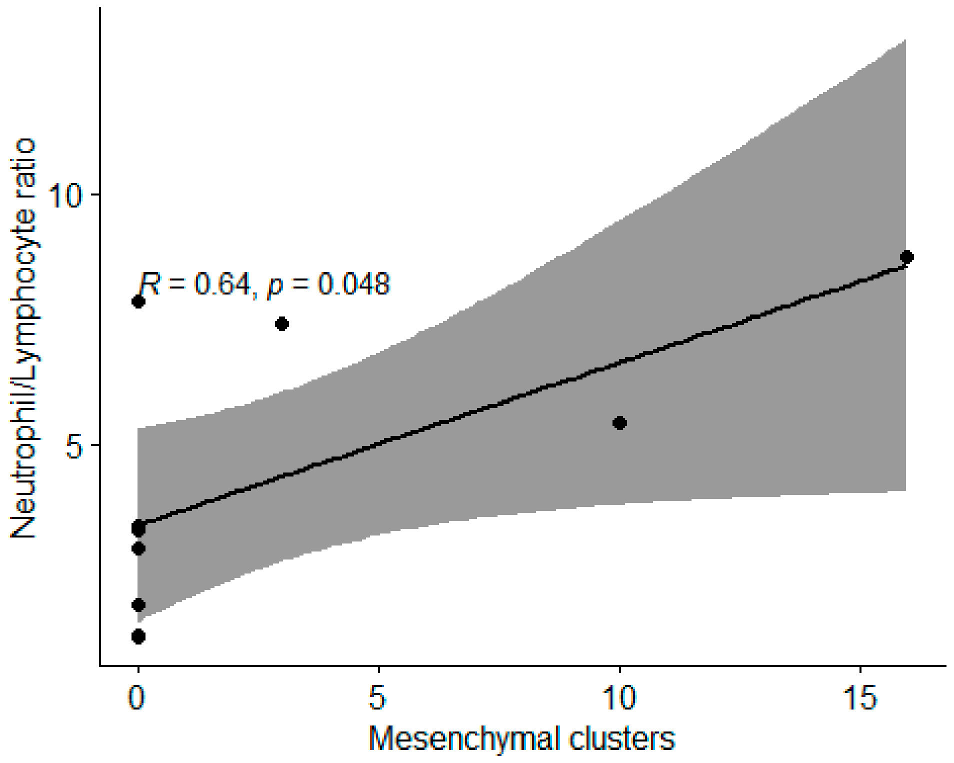

R = 0.64, p = 0.048
Neutrophil/Lymphocyte ratio
Mesenchymal clusters
10
5
0
5
10
15
